# Successful treatment of an intrathoracic bronchogenic cyst in a Holstein-Friesian calf

**DOI:** 10.1186/1751-0147-55-14

**Published:** 2013-02-19

**Authors:** Beat Berchtold, Mireille Meylan, Karine Gendron, Ute Morath, Ulrich Rytz, Beatrice Lejeune

**Affiliations:** 1 Clinic for Ruminants, Department of Clinical Veterinary Medicine, Vetsuisse Faculty, University of Berne, Berne, Switzerland; 2 Clinical Radiology, Department of Clinical Veterinary Medicine, Vetsuisse Faculty, University of Berne, Berne, Switzerland; 3 Anesthesiology Division, Department of Clinical Veterinary Medicine, Vetsuisse Faculty, University of Berne, Berne, Switzerland; 4 Clinic for Small Animals, Department of Clinical Veterinary Medicine, Vetsuisse Faculty, University of Berne, Berne, Switzerland

**Keywords:** Bronchogenic cyst, Cattle, Malformation, Thoracic surgery

## Abstract

A 5-½-month-old female Holstein-Friesian calf was presented with a history of recurring ruminal tympany and poor development. The absence of lung sounds on the right hemithorax suggested a right-sided intrathoracic pathology. Radiography and computed tomography revealed a large thin-walled cavernous lesion with a gas-fluid interface which almost completely filled the right thoracic cavity. Fluid aspirated from the lesion was clear, yellowish and odorless. These findings led to the diagnosis of a bronchogenic cyst. Thoracotomy was performed under general anesthesia. The cyst strongly adhered to the adjacent lung tissue. After removal of the free wall, the adjacent lung tissue was sealed using surgical stapling instruments, and the non-removable part of the wall was curetted and rinsed. The intensive postoperative management included antibiotic therapy, oxygen supplementation and regional lidocaine infusion. Anti-inflammatory drugs were administered for further pain control. The calf recovered well and was released from the clinic on postoperative day 11. Intra- or extrathoracic bronchogenic cysts result from abnormal budding during the embryonic development of the tracheobronchial system. Successful treatment of this calf despite the size of the lesion and the invasive character of the surgical intervention indicates that resection of bronchogenic cysts in cattle may be an option for valuable animals.

## Background

Bronchogenic cysts, also known as foregut duplication cysts, result from abnormalities in the development of the tracheobronchial system during the embryonic period [1,2]. Pulmonary development is divided into five steps, the first of which (I) begins in the embryonic stage with the formation of the laryngo-tracheal groove, followed by formation of the trachea-esophageal groove and of respiratory diverticula (days 30 to 50 of gestation in cattle). Segmental bronchi later arise from these diverticula. Subsequent steps of fetal lung development in cattle include: (II) the pseudo-glandular period (formation of all major conducting branches) from days 50 to 120 of gestation; (III) the canalicular period (enlargement of the lumina of the bronchi and bronchioles) from day 120 to day 180; (IV) the terminal sac period (days 180 to 240), where large numbers of terminal sacs bud off from the respiratory bronchioles, and finally (V) the alveolar period (from days 240 to 260 of gestation in cattle) in which the capillaries surrounding terminal sacs become associated with the alveolar epithelial cells [3].

Abnormalities in budding during the embryonic development may lead to the formation of supernumerary bronchi when continuity with the developing tracheobronchial airway is maintained, or may lead to the development of bronchogenic cysts when this connection is lost [1,3]. Bronchogenic cysts may be classified into intra- or extrathoracic cysts [4]. Intrathoracic cysts are the most common form in humans and are localized mainly in the mediastinum [5], but they may also be found within the diaphragm, pericardium or lung parenchyma [6]. Extrathoracic cysts may be found in the suprasternal notch, next to the manubrium, in the shoulder, the neck, at the base of the tongue, the infraclavicular or the chin mental region. Extrathoracic cysts may also extend into the mediastinum [4]. Correlations between bronchogenic cysts and heritability have not been described so far in the literature.

In humans, pediatric patients with symptomatic bronchogenic cysts tend to present with respiratory dysfunction due to compression of the tracheobronchial tree by the cyst. In adults however, symptoms may develop as a result of infection of a formerly asymptomatic cyst [7].

Few reports of bronchogenic cysts are available in the veterinary literature. An extrathoracic cervical bronchogenic cyst (11 × 7 × 4 cm) has been described in a calf with progressive enlargement of a mass on the ventral side of the neck [2]. A second case of bronchogenic cyst (8-10 cm in diameter) in the mediastinum and left cranial lung lobe of a German shepherd dog has been reported [8]. In both cases, the cysts could be successfully removed.

This is the first report describing the diagnostic approach, surgical treatment and follow-up of an intrathoracic bronchogenic cyst in a calf.

### Case description

A 5-½-month-old female Holstein-Friesian calf weighing 132 kg was referred to the Clinic for Ruminants of the Vetsuisse Faculty in Berne with a history of recurring ruminal tympany and poor development.

Complete clinical examination of the calf revealed a thin body condition and a rough hair coat. Body temperature was within normal limits (39.2°C; normal: 38.5-39.5°C), and examination of the cardiovascular system revealed no abnormalities (pulse rate: 96 beats/min; normal: 72 to 100 beats/min) [9]. The calf showed dyspnea with an increased respiratory rate of 60 breaths/min (normal: 20-40 breaths/min) [9] and exaggerated expiratory efforts accompanied by spontaneous unproductive cough. Increased respiratory sounds were audible over the entire lung fields upon thoracic auscultation on the left side, respiratory sounds could not be auscultated on the right side of the thorax. No nasal discharge was noticed and the breath had a neutral smell. Food intake was reduced, but swallowing, rumination and water intake were normal. The rumen was distended with an enlarged dorsal gas cap but physiologic solid and liquid phases. Rumen motility and intestinal borborygmi were present. Feces contained undigested fibers (>4 cm). Results of the examination of the other organ systems were within normal limits.

Clinical examination, in particular the absence of lung sounds on the right hemithorax, suggested a primary right-sided intrathoracic lesion and a ruminal tympany. For further characterization of the disease process, blood and rumen juice analyses, as well as thoracic radiographs were performed.

**Figure 1:**
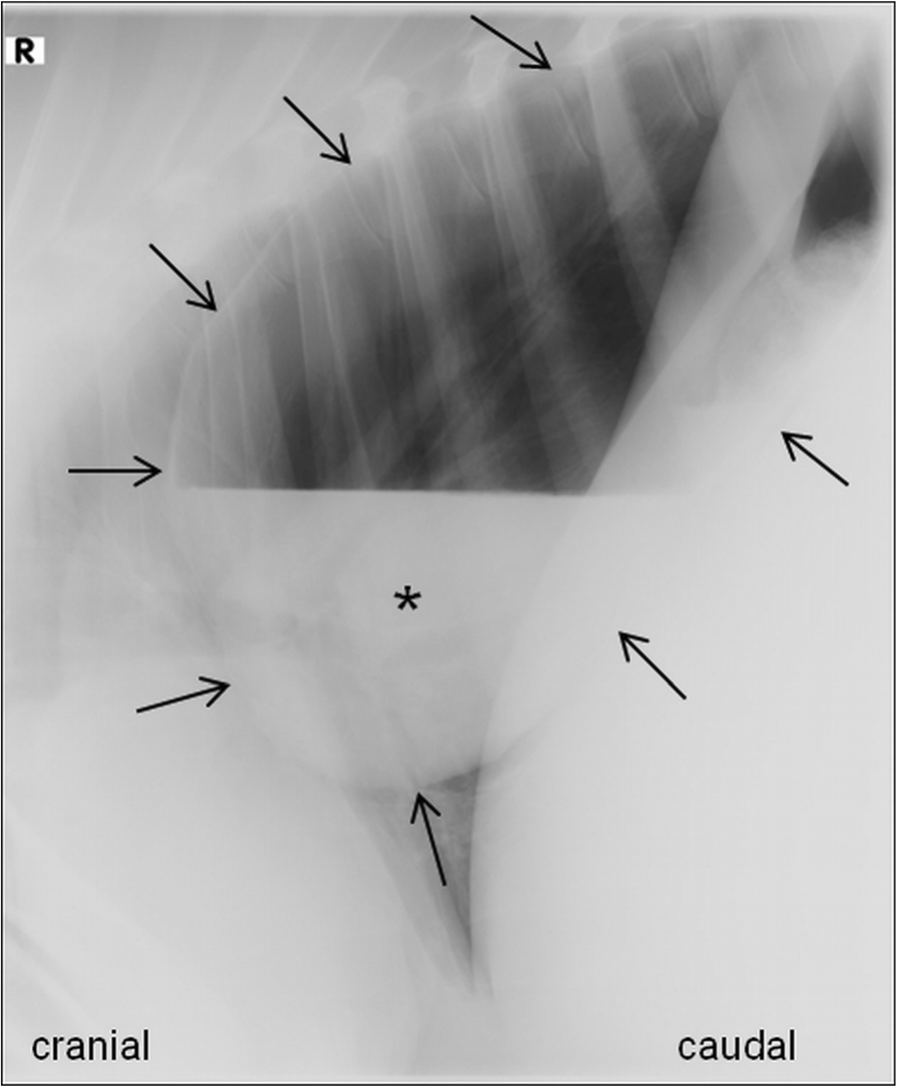
**Thoracic radiograph of a Holstein-Friesian calf with a bronchogenic cyst. **Laterolateral right-sided (R) thoracic radiograph of a 5-½-month-old female Holstein-Friesian calf with dyspnea and recurring ruminal tympany caused by a bronchogenic cyst (standing position). A cavernous, thin-walled lesion (arrows) with a gas-fluid interface is localized in the caudodorsal lung fields. Fluid within the lesion is marked by the asterix (*).

The results of the complete blood count (CBC)^a^ were within normal limits and the analysis of rumen juice revealed an inactive flora. Laterolateral thoracic radiographs on the standing animal showed a large (20×35 cm) cavernous lesion which almost completely occupied the caudodorsal thoracic cavity. The lesion was thin-walled and presented a gas-fluid interface in its ventral third (Figure 1). The visible lung parenchyma was of moderately increased radiopacity, with fine air bronchograms in the cardiophrenic angle.

**Figure 2:**
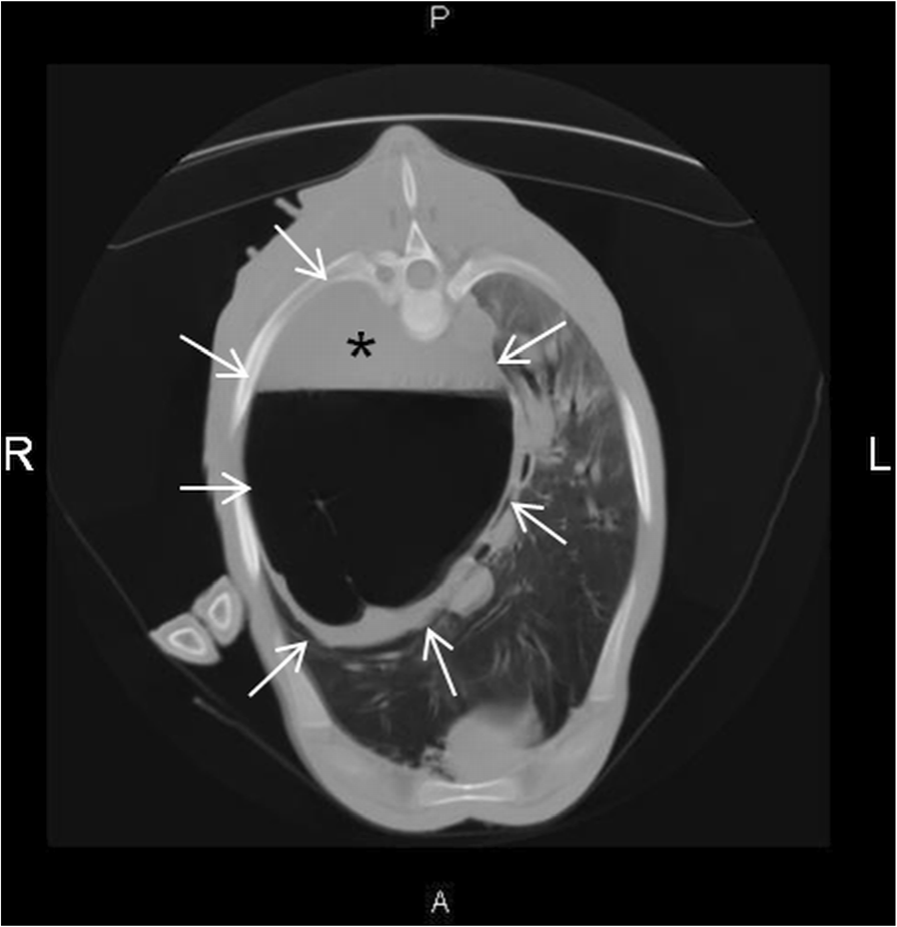
**Computed tomography of the thorax of a Holstein-Friesian calf with a bronchogenic cyst. **Computed tomography of the thorax of a 5-½-month-old female Holstein-Friesian calf with a bronchogenic cyst imaged in dorsal recumbency, transverse view caudal to the heart (image tilted 180°). A regular 2 cm thick wall (arrows) encircles the solitary cavity containing fluid (*) and gas. A multifocal alveolar lung pattern with focal bronchiectasis may be seen in the ventral right and the left caudal lobes. In the dorsal left lung fields, air bronchograms are present within diffuse and mild patchy pulmonary opacities. R right; L left; P dorsal; A ventral.

In order to better characterize the lesion, a computed tomography (CT) of the thorax was performed under general anesthesia using a 16 slice CT scanner^b^ with a peak kilovoltage (kVP) of 140 and 250 milliampere seconds (mAs). The study consisted of continuous 5 mm slices acquired with breathhold technique in dorsal recumbency. Contrast was not administered. The large cavitary lesion was localized to the right caudal hemithorax and measured approximately 21 cm in height, 17 cm in width and 19 cm in length. A regular wall, 2 cm at its thickest, encircled a single cavity containing fluid (27 Hounsfield units (HU)) in its ventral third, and gas in its dorsal portions (Figure 2). The main bronchus to the right caudal lung lobe curved around the lesion and appeared separate from it, except at the medial border of the lesion, where both structures could not be clearly delineated from one another. The bronchus to the right middle lung lobe could not be visualized. Two small thin-walled bullae could be observed cranially to the cavitary lesion and continuous to it. Additionally, a multifocal alveolar lung pattern with focal bronchiectasis in the ventral right and the left caudal lobes was seen. In the dorsal left lung fields, air bronchograms were present within diffuse and mild patchy pulmonary opacities.

The differential diagnoses for the cavitary structure in the right hemithorax included a bronchogenic cyst or an abscess. In addition, the areas of alveolar pattern suggested either focal pneumonia or compression atelectasis of the lung tissue. In the dorsal lung fields, changes were attributed to hypostasis due to dorsal recumbency during CT.

Fluid within the cyst was punctured under ultrasound guidance in the non-sedated calf. The aspirate was clear, yellowish and odorless. Its specific gravity was 1.032, total protein was 44 g/L and the white blood cell count was 0.92×10e9/L with 98% polymorphonuclear leucocytes. Few bacillary bacteria were found in the cytological analysis. Polymerase Chain Reaction (PCR)^c^ for *Mycoplasma bovis* was negative.

The clinical, radiographic and CT findings as well as the results of aspirate analysis were suggestive of a bronchogenic cyst. The recurring rumen tympany observed was considered to be secondary to esophageal compression and ructus impairment by the cyst. With the owner’s agreement, the mass was surgically removed.

The calf’s condition was reevaluated specifically prior to thoracotomy and the animal was assigned a category four out of five on the ASA Physical Status Classification System [10], indicating an elevation of the anesthetic risk. Butorphanol^d^ (0.05 mg/kg body weight (BW) IV), and xylazine^e^ (0.1 mg/kg BW IM) were administered for premedication, and the calf was preoxygenated^f^ with a face mask for 10 minutes until general anesthesia was induced with ketamine^g^ (5.3 mg/kg BW IV). After intubation in sternal recumbency, the endotracheal tube was connected to a breathing system^h^ and 100% oxygen was given to the spontaneously breathing calf. It was then placed on the surgery table in left lateral recumbency, and volume-controlled ventilation was started. General anesthesia was maintained with inhaled isoflurane^i^ in 100% oxygen and constant rate infusions of butorphanol^d^ (0.02 mg/kg BW per hour IV), lidocaine^j^ (1.8 mg/kg BW per hour IV, after an initial bolus of 1.5 mg/kg BW IV given over 10 minutes), and ketamine^g^ (0.5 mg/kg BW per hour IV). Intercostal nerve blocks were performed with a total volume of 15 ml of 2% lidocaine^j^ from the 4^th^ to the 8^th^ right rib spaces.

The skin was incised between the 6^th^ and 7^th^ rib and osteotomy of the distal aspect of the 6^th^ rib was performed with an oscillation saw^k^ to enable insertion of a Finochietto retractor^l^. The pleura was incised and exploration of the pleural cavity was performed. The cyst was embedded between the caudal part of the cranial lung lobe and the middle lobe, and adhered to the adjacent lung tissue, which prevented its *in toto* removal. The cyst wall was incised and the fluid aspirated. The free part of the wall was removed using surgical stapling instruments^m^ to seal the adjacent lung tissue. The inside of the non-removable part of the wall was rinsed with sterile sodium-chloride solution^n^ and curetted. A chest tube^o^ was placed through the 8^th^ intercostal space and connected to a silicone reservoir^p^ providing negative pressure for 48 hours. The pleura and the intercostal muscles were adapted and sutured with resorbable material^q^. The 6^th^ rib was aligned and stabilized to the 5^th^ and 7^th^ rib using absorbable suture material^q^ in a circumcostal pattern. Muscles, subcutis and skin were closed routinely after installation of a perforated flexible plastic tube^r^ with a diameter of 3 mm between the adapted muscle layers to instill lidocaine^j^ for postoperative analgesia.

Postoperative management consisted antibiotic therapy (penicillin G^s^, 30’000 IE/kg BW IV, q8h, for 12 days, and danofloxacin^t^, 1.25 mg/kg BW, q24h IV, for 5 days) and oxygen insufflation through a nasal tube for 24 hours. Regional lidocaine^j^ infusion (1 mg/kg BW every 2 hours) was administered through the flexible plastic tube placed at surgery and anti-inflammatory agents were administered for further pain control (flunixine meglumine^u^, 2.2 mg/kg BW IV, q24h for two days, followed by ketoprofen^v^, 4.5 mg/kg BW PO, q24h, for the next five days), as well as butorphanol^d^ (0.01 mg/kg BW IV, q2h for the first 36 hours after surgery). Arterial blood gas measurements were used to monitor lung function.

Twenty-four hours after surgery, normal arterial blood gas parameters combined with the calf’s good general condition allowed discontinuation of oxygen supplementation. The chest tube^p^ and the perforated flexible plastic tube^r^ for lidocaine instillation were removed 48 hours after surgery.

The calf recovered steadily and was released from the clinic 11 days postoperatively. At this time, the respiratory parameters were satisfactory, characterized by an increased respiratory rate of 60 breaths/min [9] and wheezes over the left as well as over the right lung field. These wheezes were considered to be either a consequence of re-ventilation of the previously compressed lung tissue, and/or due to focal pneumonia in this area considering the CT findings on the right side. The wheezes over the left lungs were considered to be a late consequence of surgery, i.e. of intubation and mechanical ventilation as well as of compression due to prolonged recumbency on the left side during the surgical intervention.

No more spontaneous unproductive cough could be heard. The arterial blood gas analysis showed an oxygen saturation of 93.7% (reference range: >98%) and a partial oxygen pressure of 73.0 mmHg (reference range: 95 – 105 mmHg) [11]. The calf showed no signs of dyspnea. Antibiotic therapy was continued for 9 days after release from the clinic with procaine penicillin G^w^ (30’000 IE/kg BW IM, q24h). Ruminal tympany was not observed after surgery.

**Figure 3:**
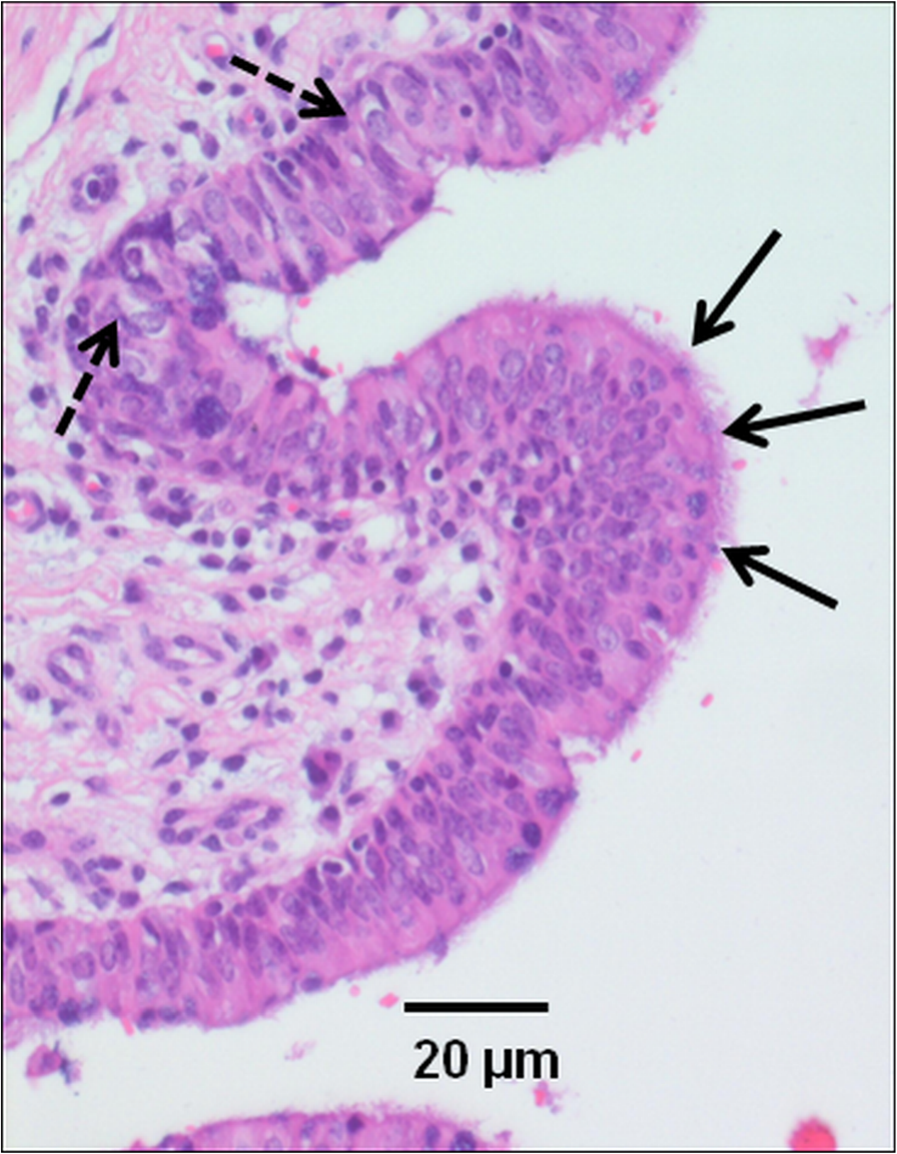
**Histological section of the bronchogenic cyst wall of a Holstein-Friesian calf. **Histological section of the bronchogenic cyst wall of a 5-½-month-old female Holstein-Friesian calf. Histology revealed a ciliated pseudostratified epithelium (arrows) and goblet cells (spotted arrows). Hematoxylin & Eosin (HE) staining; bar = 20 μm.

Histological findings of the extirpated cyst revealed a ciliated pseudostratified epithelium, glandular structures, goblet cells and granulation tissue. The wall was infiltrated with inflammatory cells (Figure 3).

The calf returned to the clinic for reevaluation 3 months after surgery. Its general condition was good, as confirmed by a weight gain of 133 kg since its release from the clinic, as well as by an appropriate size and muscle mass for its age. The respiratory rate was still above normal (48 breaths/min), and the respiratory sounds were slightly increased bilaterally over the entire lung fields, but no pathologic sounds such as wheezes or crackles were heard.

**Figure 4:**
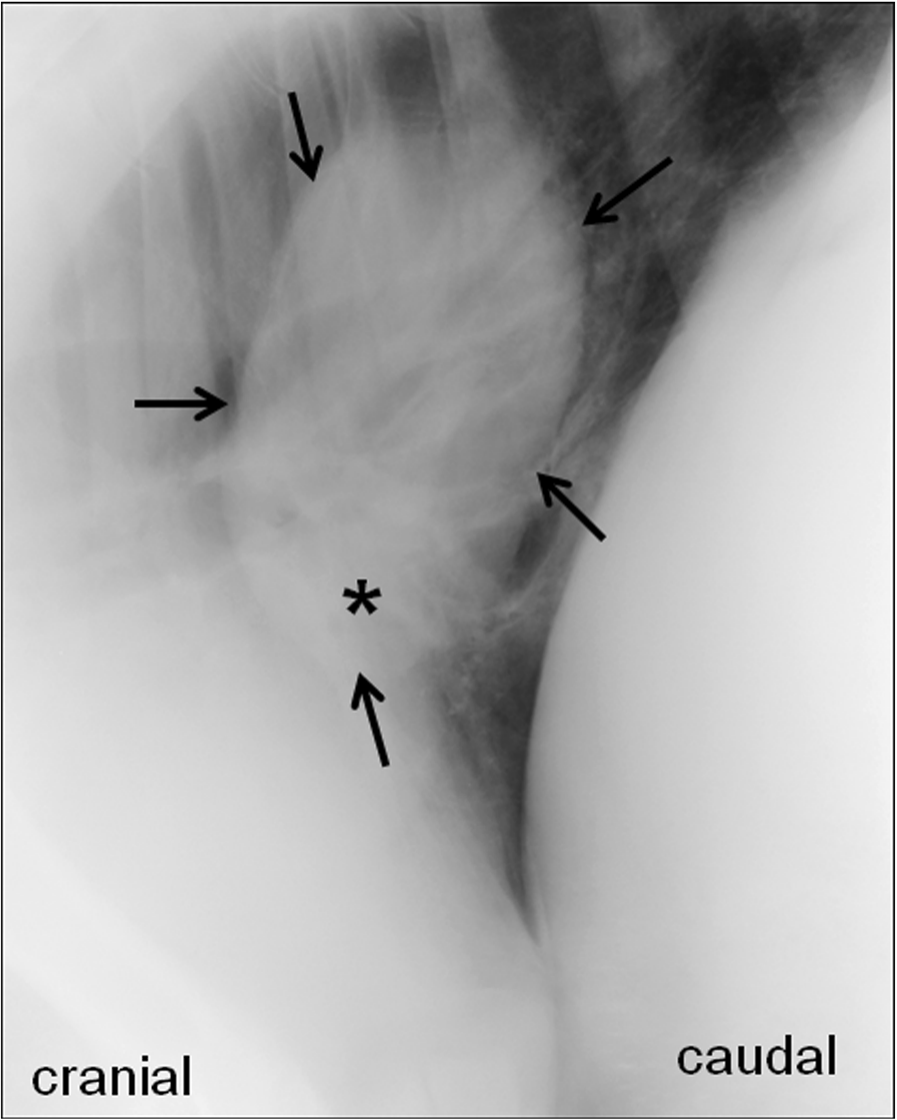
**Radiologic control after partial surgical removal of an intrathoracic bronchogenic cyst in a calf. **Radiologic control of the thorax three months after partial surgical removal of an intrathoracic bronchogenic cyst in a Holstein-Friesian calf. The laterolateral right-sided thoracic radiograph in standing position shows a radiopaque structure (arrows) with little fluid content (*) at the former location of the cyst. The radiopacity of the lung tissue is increased ventrally to the lesion.

Thoracic radiographs revealed a homogenous structure of soft-tissue opacity at the former location of the cyst and increased radiopacity of the adjacent tissues (Figure 4)*.* Because of the satisfactory clinical evolution and because a further intervention was not envisaged for this animal, no further examinations were undertaken to differentiate scar tissue from possible recurrence of a fluid-filled cyst.

The development and the general condition of the calf were satisfying and it calved for the first time at the age of 24 months.

## Discussion

In the presented case, the Holstein Friesian calf was referred to the Clinic for Ruminants of the Vetsuisse Faculty in Berne for clinical examination of recurring ruminal tympany and poor development.

Bronchogenic cysts may present clinically with progressive dyspnea and decrease or absence of lung sounds on the affected side, as do also other intrathoracic space-occupying lesions like abscesses, seromas or neoplastic masses. Other clinical signs vary according to the specific location of the lesion and may include ruminal tympany (due to compression of the esophagus or vagal dysfunction) and congestion of the jugular veins as a result of compression by the space-occupying masses [6,12]. In the present case, rumen tympany was likely caused by compression of the esophagus as this sign disappeared after surgery. Vagal dysfunction would not have been expected to resolve after excision of the cyst.

Although clinical examination and radiography revealed enough details to reach a suspicion of bronchogenic cyst in the present case, CT was helpful to better evaluate the cyst’s dimensions and its relation to the surrounding structures prior to surgery. Assessment of the thorax by CT is used in humans for differentiation between bronchogenic cysts, congenital lobar emphysema, and congenital cystic adenomatoid malformation [13]. As cyst fluid contains mucus and occasionally calcium, its CT density is variable, but it is often higher than that of water. Contrast enhancement of the cyst wall may occur in the face of infection [14]. Thoracic CT is also routinely performed in dogs [15], but its use is still limited in cattle due to the size of the animals and to financial constraints.

Despite CT’s sensitivity, histopathologic examination of the excised material is still considered necessary to confirm a diagnosis of bronchogenic cyst [1]. Bronchogenic cysts are typically lined with a ciliated pseudostratified columnar to cuboidal respiratory epithelium [2]. Other typical histological features of bronchogenic cysts are a fibromuscular wall and seromucous bronchial glands. The presence of cartilage is highly characteristic of bronchogenic cysts [1,12,14].

In the present case, no cartilage was found in the tissues submitted for histology. Nevertheless, the presence of a ciliated pseudostratified epithelium, of glandular structures and goblet cells correlates well with published descriptions [1,4,14].

Bronchogenic cysts usually do not communicate with the tracheobronchial tree and are filled with serous or mucoid fluid [3,14]. An air-fluid level may be the consequence of iatrogenic intervention (puncture) or infection, which usually occurs secondary to communication with the tracheobronchial tree [14]. In the present case, fluid puncture was performed after the CT and can thus be excluded as a cause for the presence of air or gas in the cyst. Although neither CT nor surgery revealed a clear communication to the tracheobronchial tree, the presence of a thin communication cannot be excluded with certainty. Alternatively, the gas may have been produced by the bacillary bacteria detected in the aspirated fluid, although analysis of the punctured fluid revealed a low white blood cell count and only few bacteria.

Therapeutic options for bronchogenic cysts are limited. Complete surgical excision is recommended and should be curative, whereby aspiration of the cyst fluid seems to be an inadequate option as secretions likely will reaccumulate. In case of extrathoracic lesions without clinical signs, surgery may be performed for cosmetic reasons [15]. Although controversial, in humans, surgical excision has been recommended in asymptomatic patients in order to prevent complications such as lung abscesses. In fact, secondary cyst infection is estimated to occur in 20% of cases [7]. Other reported complications include cyst rupture with formation of pneumothorax or respiratory distress as well as pulmonary hemorrhage and hemoptysis [2,4,5,7,12]. In the present case, complete excision of the cyst was not possible. However, removing the free part of the cyst wall allowed curing the clinical signs, in particular the recurrent ruminal tympany due to esophageal compression. No significant volume of fluid was observed in the remnants of the cyst on the control radiographs performed 3 months after surgery, and no clinical signs of recurrence had been observed 1.5 years after partial removal of the cyst.

## Conclusion

Intrathoracic bronchogenic cysts may cause ruminal bloat in cattle, and they may be successfully treated, despite thoracotomy in calves being an invasive and expensive operation dependent on sophisticated anesthesiology equipment and surgical knowledge. In the light of the present patient’s uneventful recovery despite the size of the lesion and the invasive character of the surgical intervention, excision of bronchogenic cysts in cattle may be an option for selected cases. However, as the cyst could not be resected *in toto*, it cannot be excluded that later complications may affect this animal’s productive life. Furthermore, no information is available on potential heritability of bronchogenic cysts in cattle.

## Consent

Written informed consent was obtained from the patient’s owner in keen for publication of this report and any accompanying images.

## Endnotes

^a^Advia 120, Siemens Medical Solutions Diagnostics, Erlangen, Germany

^b^Brilliance, Philips Medical Systems, Eindhoven, Netherlands

^c^7500 real-time PCR system, Applied Biosystems International Inc., Rotkreuz, Switzerland

^d^Morphasol®-10 ad.us.vet, Dr. E. Graeub AG, Bern, Switzerland

^e^Xylazin Streuli ad.us.vet, Streuli Pharma AG, Uznach, Switzerland

^f^Sauerstoff O2, Carbagas AG, Gümligen, Switzerland

^g^Ketanarkon 100 ad.us.vet., Streuli Pharma AG, Uznach, Switzerland

^h^Fabius®-CE, Dräger Medical Schweiz AG, Liebefeld, Switzerland

^i^AttaneTM Isoflurane ad.us.vet., Provet AG, Lyssach bei Burgdorf, Switzerland

^j^Lidocain 2% Streuli ad.us.vet., Streuli Pharma AG, Uznach, Switzerland

^k^Oscillating Saw Attachment with variable deflection, Synthes GmbH, Oberdorf, Switzerland

^l^Finochietto rip spreader, Aesculap Inc., Center Valley, USA

^m^Surgical Proximate linear cutter 75 mm, Ethicon, Medical&Medical GmbH, Norderstedt, Germany

^n^Natrium chloratum 0.9% “Bichsel”, Dr. G. Bichsel AG, Interlaken, Switzerland

^o^24CH, Mallinckrodt Medical, Athlone, Ireland

^p^Fortune Medical Instrument Corp., Jungjeng E. Rd. Danshuei Jen Taipei Hsien 251, Taiwan

^q^Polydioxanon (PDSII), Ethicon, Medical&Medical GmbH, Norderstedt, Germany

^r^Katheter-Rüsch, Wirtschaftsgenossenschaft deutscher Tierärtzte eG, Garbsen, Germany

^s^Penicillin Natrium Streuli ad.us.vet., Streuli Pharma AG, Uznach, Switzerland

^t^Advocid® 2.5% ad.us.vet, Pfizer AG, Zürich, Switzerland

^u^Flunixine® Biokema ad.us.vet, Biokema SA, Crissier, Switzerland

^v^Dolovet ad.us.vet., Dr. E. Graeub AG, Bern, Switzerland

^w^Procacillin ad.us.vet., Veterinaria AG, Pfäffikon, Switzerland

## Abbreviations

BW: Body weight; CBC: Complete blood count; CT: Computed tomography; HU: Hounsfield units; PCR: Polymerase chain reaction; kVP: Peak kilovoltage; mAs: Milliampere second; g/L: Gram per liter.

## Competing interests

The authors declare that they have no competing interests.

## Authors’ contributions

BB did the clinical examination, assisted the operation and followed up the post-operativ management and is the main author of the paper. KG carried out the diagnostic imaging. UM performed the general anesthesia. UR was the main surgeon for thoracotomy. MM supervised clinical management and editing of the report. BL supported and controlled the clinical examination as well as the post-operative management and assisted the operation. Furthermore she drafted and helped to write the final version. All authors read and approved the final manuscript.
